# Pneumothorax and Pneumomediastinum Complicating Organophosphate Poisoning: A Case Series of Complications Less Understood

**DOI:** 10.7759/cureus.22481

**Published:** 2022-02-22

**Authors:** Nidhi Kaeley, Ankita Kabi, Hari Prasad, Alok Raj, Anirban Ghosh Hazra

**Affiliations:** 1 Emergency Medicine, All India Institute of Medical Sciences, Rishikesh, IND; 2 Emergency Medicine (Anaesthesiology), All India Institute of Medical Sciences, Rishikesh, IND

**Keywords:** sepsis, pneumothorax, pneumomediastinum, organophosphates, acute respiratory distress syndrome

## Abstract

Organophosphate compounds are used as insecticides in agricultural and domestic settings throughout the world. Acute organophosphorus (OP) poisoning is a major public health issue. Early diagnosis of OP poisoning and prompt atropinization can save lives. Respiratory failure may occur in patients with OP poisoning for many reasons, including aspiration of gastric contents, excessive secretions, pneumonia, and sepsis complicating acute respiratory distress syndrome. Till date, however, spontaneous pneumothorax and pneumomediastinum have not been reported in cases of OP poisoning. This report presents two similar cases of OP poisoning in which spontaneous pneumothorax and pneumomediastinum developed following OP ingestion.

## Introduction

Organophosphate compounds are widely used as insecticides, herbicides, and pesticides. Organophosphorus (OP) poisoning can occur by accidental or deliberate exposure [1]. According to recent data from the National Crime Bureau of India, the mortality rate for patients with pesticide ingestion was 26.6% in 2017 [2]. Common organophosphate compounds are malathion, parathion, fenthion, diazinon, dimethoate, and chlorpyrifos [3]. The most common cause of mortality after OP poisoning is respiratory failure and lung injury. Respiratory complications can manifest during an acute cholinergic crisis, as intermediate syndrome, or with delayed OP poisoning effects [4]. This report presents two similar but rare cases of pneumothorax and pneumomediastinum following exposure to organophosphate compounds.

## Case presentation

Case 1

A 78-year-old man with no known comorbidities or medication history was transferred to the emergency department of a tertiary care institute in Uttarakhand after ingestion of 50-mL chlorpyrifos and cypermethrin at his home. After ingestion, he had multiple episodes of nonbloody, nonbilious vomiting and was immediately taken to the local hospital. Gastric lavage was performed there, and then the patient was transferred to the emergency department and arrived in an unconscious state. No fever, cough, or seizures were observed on presentation. No history of any associated trauma.

On examination, the patient was unconscious. His vital signs were blood pressure 90/60 mmHg, heart rate 104 beats/minute, and respiratory rate 24 breaths/minute. His Glasgow coma scale score was E2V1M4. Etomidate and rocuronium were administered, and the patient was intubated with these settings: volume control assist control (VCAC) mode, fraction of inspired oxygen (FiO_2_) 100%, tidal volume (Vt) 360 mL, respiratory rate 16 breaths/minute, and positive end-expiratory pressure (PEEP) 5 cm H_2_O; oxygen saturation was 60% on room air and 88% with a 10-L oxygen facemask.

Respiratory system examination showed bilateral basal crackles and decreased breath sounds on the right side of the chest. A lung ultrasound showed the B profile with lung sliding and the “shred” sign bilaterally. Central nervous system examination showed bilateral 1-mm pinpoint pupils not reactive to light. Other system examinations were normal.

Laboratory investigations revealed normocytic normochromic anemia with neutrophilic leukocytosis and mild transaminitis as shown in Table 1 (Case 1).

**Table 1:** Blood test results ALT: Alanine aminotransferase; AST: Aspartate aminotransferase; ALP: Alkaline phosphatase; INR: International normalised ratio

Parameters	Case 1	Case 2	Reference range
Hemoglobin	15.8 g/dL	13 g/dL	12–15 g/dL
Red blood cell count	3.68 × 10^6^/mcL	4.64 × 10^6^/mcL	3.8–5.2 × 10^6^/mcL
White blood cell count	15.08 × 10^3^/mcL	12.09 × 10^3^/mcL	4–11 × 10^3^/mcL
Platelets	193 × 10^3^/mcL	255 × 10^3^/mcL	150–400 × 10^3^/mcL
Total bilirubin	1.2 mg/dL	0.66 mg/dL	0.3–1.2 mg/dL
Direct bilirubin	0.6 mg/dL	0.37 mg/dL	0–0.2 mg/dL
ALT	49 U/L	47 U/L	0–35 U/L
AST	63 U/L	72 U/L	0–35 U/L
ALP	330 U/L	115 U/L	30–120 U/L
Serum albumin	3.6 g/dL	4.4 g/dL	3.5–5.2 g/dL
Urea	42 mg/dL	29 mg/dL	17–43 mg/dL
Creatinine	1.27 mg/dL	0.2 mg/dL	0.55–1.02 mg/dL
Sodium	139 mEq/L	135 mEq/L	136–146 mEq/L
Potassium	4 mEq/L	3.5 mEq/L	3.5–5.1 mEq/L
Calcium	8.1 mg/dL	7.7 mg/dL	8.8–10.6 mg/dL
Prothrombin time	17.3 sec	21.4 sec	13.6 sec
INR	1.29	1.61	0.90–1.20

In the emergency department, the patient was atropinized with 30 mg atropine and atropine infusion was initiated at 6 mg/h. Ceftriaxone 1 g twice daily and clindamycin 600 mg twice daily were also started.

**Figure 1:**
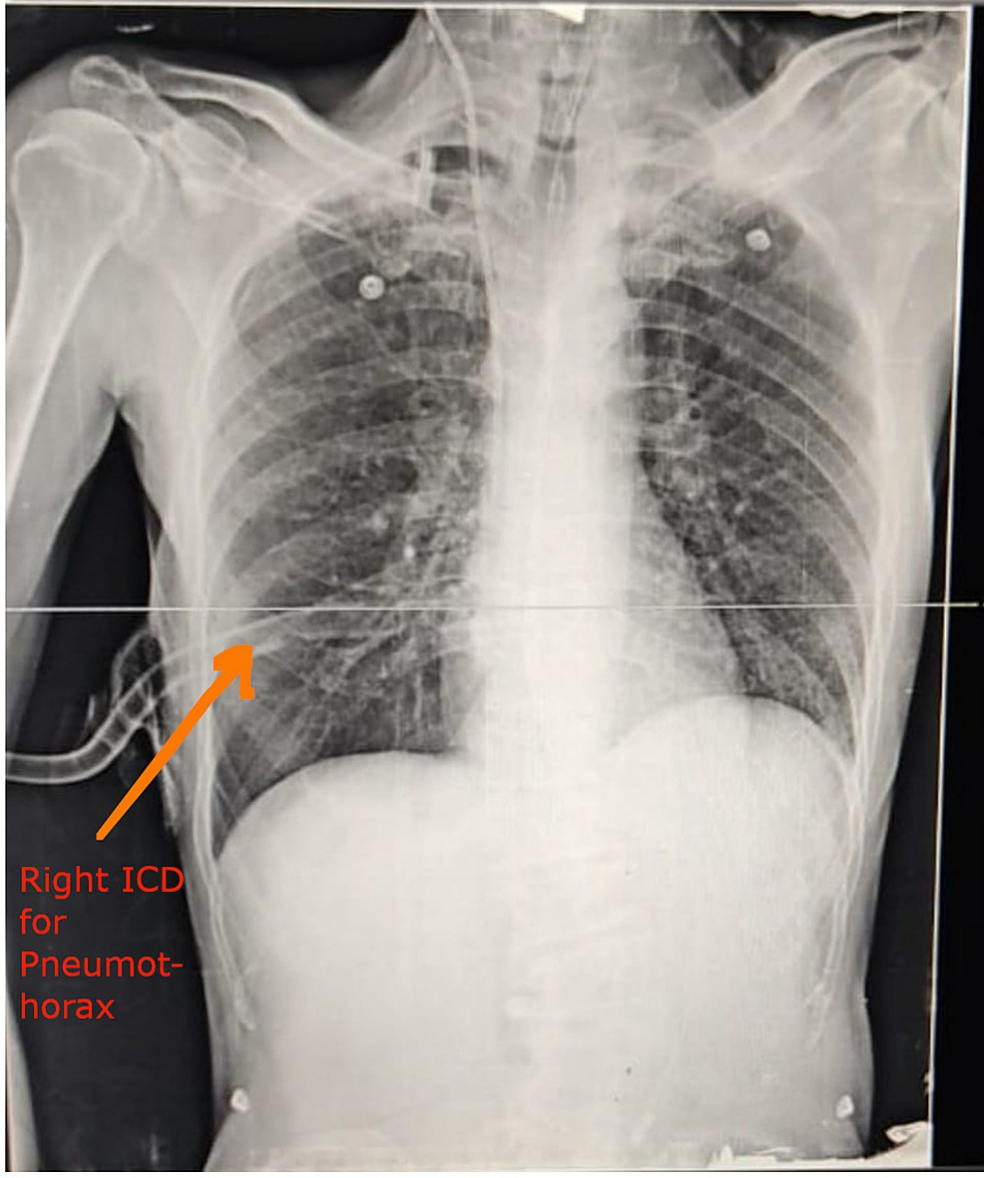
Chest radiography showing right intercostal drainage tube in situ for pneumothorax (Case 1) ICD: Intercostal drainage tube

On day two, the ventilator settings were as shown in Table 2. On day three, plateau pressure (36 cm H_2_O) and peak pressure (42 cm H_2_O) suddenly started rising. A lung ultrasound showed the “lung point” sign with absent sliding on the right side. Chest radiography displayed right-sided pneumothorax for which intercostal tube was inserted as shown in Figure 1. High-resolution computed tomography (HRCT) of the thorax revealed right-sided pneumothorax with 5-mm pneumomediastinum.

**Table 2:** Ventilator settings in volume control assist control mode during hospital stay (Case 1)

Day	Fraction of inspired oxygen, %	Tidal volume, mL	Respiratory rate, breaths/min	Positive end-expiratory pressure, cm H_2_O	Plateau pressure, cm H_2_O	Peak pressure, cm H_2_O	
1	100	360	16	5	20	26	
2	50	360	14	5	18	28	
3	50	360	14	5	36	42	

A thoracostomy tube was placed for the right-sided pneumothorax. Despite all these measures, the patient’s oxygen saturation and blood pressure did not improve; noradrenaline and vasopressin were started. On day three, the patient died.

Case 2

A 20-year-old woman with no known comorbidities or medication history was transferred to the emergency department of a tertiary care institute in Uttarakhand after ingestion of 30 mL chlorpyrifos and cypermethrin at her home. After ingestion, she had multiple episodes of non-bloody, non-bilious vomiting, and became drowsy. She also experienced shortness of breath, increased lacrimation, salivation, and involuntary passage of stools. There was no history of fever, cough, or trauma. She was immediately taken to the local hospital where gastric lavage was performed, and then the patient was transferred to the emergency department.

On examination, the patient was drowsy. The following vital signs were evaluated: blood pressure 120/70 mmHg, heart rate 110 beats/minute, and respiratory rate of 26 breaths/minute, with oxygen saturation 68% on room air and 98% with 4-L oxygen nasal prongs. Her Glasgow coma scale score was 9. Respiratory system examination showed bilateral basal crackles. Pupils were 2 mm and sluggishly reactive to light. Other system examinations were normal. Laboratory investigations revealed anemia with neutrophilic leukocytosis, mild transaminitis, and an increased international normalized ratio as shown in Table 1 (Case 2).

In the emergency department, the patient was atropinized with 12-mg atropine and started on atropine infusion at 2.4 mg/h. She was initially placed on noninvasive ventilation due to increased work of breathing (FiO2 100%, PEEP 6 cm H_2_O, pressure support 8 cm H_2_O).

On day two, the patient was intubated with etomidate and rocuronium because of persistent desaturation. Ventilator settings were VCAC mode, FiO_2_ 100%, Vt 380 mL, respiratory rate 14 breaths/min, and PEEP 5 cm H_2_O. Pralidoxime 2 g twice daily, ceftriaxone 1 g twice daily, and clindamycin 600 mg twice daily were started. Intubation ventilator settings are shown in Table 3.

**Table 3:** Ventilator settings in volume control assist control mode during hospital stay (Case 2)

Day of intubation	Fraction of inspired oxygen, %	Tidal volume, mL	Respiratory rate, breaths/min	Positive end-expiratory pressure, cm H_2_O	Plateau pressure, cm H_2_O	Peak pressure, cm H_2_O	
1	100	380	14	5	22	28	
2	50	380	16	5	24	29	
3	50	380	14	5	39	48	

**Figure 2:**
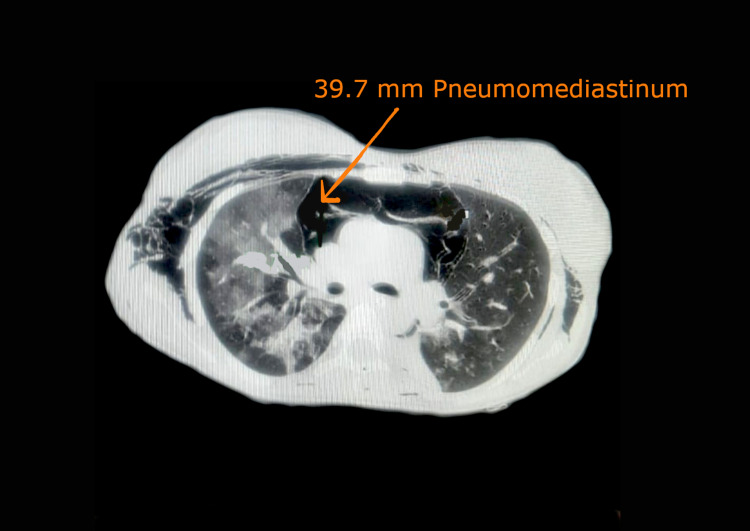
High-resolution computed tomography of the thorax shows bilateral pneumothorax with left parasternal 39.7 mm pneumomediastinum (Case 2)

On day three, plateau pressure (39 cm H_2_O) and peak pressure (48 cm H_2_O) suddenly started rising. A lung ultrasound showed the lung point sign with absent lung sliding bilaterally. On day three, HRCT of the thorax revealed bilateral pneumothorax with left parasternal 39.7 mm pneumomediastinum as shown in Figure 2. The bilateral pneumothorax was managed conservatively. Pigtail insertion was performed for pneumomediastinum drainage.

On day 12, the patient was extubated. On day 19, repeat HRCT chest revealed resolution of pneumomediastinum and pneumothorax. On day 22, the pigtail drain was removed. On day 25, she remained stable and maintained normal vital signs on room air, and was discharged. The patient is now asymptomatic with regular follow-up in the general medicine outpatient department.

## Discussion

OP poisoning is a major public health concern. A recent systematic review and a subsequent large Indian study showed that the annual mortality rate for OP poisoning is around 350,000 people in cases of intentional poisoning [5,6]. The main modes of exposure are topical, inhalation, or ingestion of organophosphate compounds [1]. Common organophosphate insecticides carry either two methyl (e.g., demeton-S-methyl, dichlorvos, dimethoate, malathion) or two ethyl (e.g., chlorpyrifos, diazinon, parathion) ester groups attached to the phosphorus atom [7]. The organophosphate compounds inhibit acetylcholine esterase at muscarinic and nicotinic synapses by deposition of the phosphorous group at the active site of the enzyme [8].

Nicotinic manifestations include muscle fasciculations and mydriasis, whereas muscarinic manifestations include excessive salivation, miosis, and diarrhea. The most frequent signs reported are miosis, vomiting, hypersalivation, respiratory distress, abdominal pain, depressed level of consciousness, and muscle fasciculations [9].

Most deaths after OP poisoning occur acutely due to hypoxia because of a combination of peripheral acute cholinergic effects and central apnea, which can be worsened by seizures (particularly with OP nerve agents). Other deaths occur later from cardiovascular distributive shock, neuromuscular junction dysfunction, and recurrent cholinergic toxicity [4]. Complications of OP poisoning can be cardiovascular, respiratory, neurologic, gastrointestinal, renal, or metabolic [10].

Respiratory complications of OP poisoning can occur during, or as a consequence of, the acute cholinergic crisis, delayed neuromuscular dysfunction, and recurrent cholinergic toxicity [4]. The acute cholinergic syndrome affects the local airway, central nervous system, and neuromuscular junction, in addition to causing alveolar fluid, bronchorrhea, acute respiratory distress syndrome (ARDS), or aspiration pneumonitis. During the acute cholinergic crisis, respiratory failure can occur from local pulmonary muscarinic effects (e.g., bronchoconstriction, bronchorrhea, and alveolar edema) or central depression of the respiratory center [11]. Pneumothorax and pneumomediastinum are observed in paraquat poisoning, with an incidence of one in 12,000 cases of hospital admission, whereas no studies have reported spontaneous pneumothorax and pneumomediastinum as a complication of acute cholinergic crisis [12].

Potential etiologies of pneumothorax and pneumomediastinum are intubation-induced barotrauma. However, we consistently maintained the plateau pressure and peak pressure less than 30 cm H2O in the two cases presented in our report. Notably, for both cases, a sudden rise occurred on day three in both these pressures, which can be due to an underlying Macklin-like phenomenon. Associated with OP poisoning, the Macklin-like phenomenon involves the destruction of type II pneumocytes that results in secondary atelectasis, rupture, and emphysematous changes, which consequently cause an increase in surface tension and the subsequent formation of peripheral or subpleural bullae that rupture, which then causes pneumothorax and escape of the gas to dissect along vascular sheaths and connective tissue planes in the mediastinum, ultimately resulting in pneumomediastinum [13]. Other explanations may be ARDS or aspiration pneumonitis, both of which can independently produce spontaneous pneumothorax or pneumomediastinum during the acute phase [14,15]. Laniee et al., in their animal experiment, found that OP poisoning causes exudative infiltration by breaking down the alveolar epithelial-endothelial barrier and that this effect was the beginning of ARDS [16]. Another respiratory complication is paralysis of proximal muscles that particularly affects the muscles of respiration, known as type II paralysis or intermediate syndrome [17].

Management includes resuscitation with oxygen and careful fluid management plus the intravenous administration of doubling doses of atropine to patients with cholinergic features and gastric lavage [18]. Other treatment modalities include oximes, inhaled beta-2 agonists, parenteral or aerosolized human or recombinant butyryl choline esterase, and fresh frozen plasma [19]. In cases that require ventilation, the use of a lung-protective ventilation strategy is recommended per the Acute Respiratory Distress Syndrome Clinical Network (ARDSnet) protocol while maintaining plateau pressures less than 30 cm H_2_O [20].

## Conclusions

OP poisoning is a serious condition that needs urgent diagnosis and management. In addition to the usually reported complications, pneumothorax and pneumomediastinum should also be kept in mind in the list of respiratory complications, which, if identified early, can save the life of the patient. Repeated lung ultrasonography and a careful look at the pressures can provide a clue to this complication of OP poisoning.
